# The role of the mitochondria and the endoplasmic reticulum contact sites in the development of the immune responses

**DOI:** 10.1038/s41419-017-0237-7

**Published:** 2018-02-28

**Authors:** Denis Martinvalet

**Affiliations:** Department of Cell Physiology and Metabolism, Geneva Medical School, 1211 Geneva, Switzerland

## Abstract

Mitochondria and endoplasmic reticulum (ER) contact sites (MERCs) are dynamic modules enriched in subset of lipids and specialized proteins that determine their structure and functions. The MERCs regulate lipid transfer, autophagosome formation, mitochondrial fission, Ca^2+^ homeostasis and apoptosis. Since these functions are essential for cell biology, it is therefore not surprising that MERCs also play a critical role in organ physiology among which the immune system stands by its critical host defense function. This defense system must discriminate and tolerate host cells and beneficial commensal microorganisms while eliminating pathogenic ones in order to preserve normal homeostasis. To meet this goal, the immune system has two lines of defense. First, the fast acting but unspecific innate immune system relies on anatomical physical barriers and subsets of hematopoietically derived cells expressing germline-encoded receptors called pattern recognition receptors (PRR) recognizing conserved motifs on the pathogens. Second, the slower but very specific adaptive immune response is added to complement innate immunity. Adaptive immunity relies on another set of specialized cells, the lymphocytes, harboring receptors requiring somatic recombination to be expressed. Both innate and adaptive immune cells must be activated to phagocytose and process pathogens, migrate, proliferate, release soluble factors and destroy infected cells. Some of these functions are strongly dependent on lipid transfer, autophagosome formation, mitochondrial fission, and Ca^2+^ flux; this indicates that MERCs could regulate immunity.

## Facts

1. MERCs are dynamic functional modules enriched in a subset of lipids and specialized proteins that dictate both their structures and functions.
2. The activation of NLRP3 inflammasome and of MAVS-dependent antiviral response takes place at the MERCs, suggesting that these contact sites play a critical role in innate immunity.
3. MERCs play an important role in cellular Ca^2+^ homeostasis by regulating ER to mitochondrial Ca^2+^ shuttling. Since Ca^2+^ signaling is essential for lymphocyte activation, this suggests that MERCs may regulate the activation of these cells during adaptive immunity.
4. MERCs regulate both autophagy and mitochondrial fission; both processes are directly linked to antigen presentation and leukocyte migration, respectively.
5. MERCs are altered in glioma stemlike cells and consequently affect glioma stemlike cell surface glycan expression, as well as susceptibility to cytotoxic lymphocytes.

## Open questions

1. What is the dynamic of the MERCs in immune cells?
2. What are the specific characteristics of immune cell MERCs?
3. Can MERCs be targeted for immune modulation?
4. MERCs are altered in glioma stemlike cells; is this a new feature of cancer stem cells?

## Introduction

The endoplasmic reticulum (ER), the largest organelle in the cell, is essential for protein synthesis, folding, maturation, transport, lipid synthesis and calcium (Ca^2+^) homeostasis. The dysregulation of the ER protein folding function triggers ER stress leading to apoptosis if not resolved^1–4^. This tentacular ER interacts with other organelles to form membrane contact sites. At the mitochondria and ER membrane contact sites (MERCs) the two organelles are ~15–50 nm apart^3,5–13^. The portion of membranes involved in these interactions defines the mitochondrial associated membranes (MAMs), which account for 5–20% of the mitochondrial network^3,13,14^. MERCs are enriched in a subset of lipids and specialized proteins that dictate both their structures and functions^3,4,12,15^. Moreover, the MERCs density, length and thickness depend on the cellular metabolic state and stress level, indicating that MERCs are dynamic and regulated functional units^5,13,16,17^. Interestingly, the MERCs are crucial for lipid transfer, initiation of autophagosome formation, determination of the mitochondrial fission site, ER-mitochondria Ca^2+^ shuttling and apoptosis^11,14,18–28^. It appears that MERCs regulate essential functions of cells biology and therefore organ physiology, among which the immune system stands by its crucial defense function.

The immune system, through its fundamental ability to distinguish self (including beneficial commensal microbiota) from non-self is able to robustly eliminate pathogenic entities and toxic molecules while preserving the integrity of the surrounding host tissues^29–33^. To achieve its protective function, the immune system relies on anatomical physical barriers (the skin and the mucosa lining the respiratory, gastrointestinal and urogenital tracts) and a subset of hematopoietically derived cells, called leukocytes (macrophages, dendritic cells, mast cells, neutrophils, eosinophils, and natural killer (NK) cells)^29,30^. Soluble factors, such as the complement system, pentraxins, collectins and the defensins antimicrobial peptides complete this arsenal^29,30^.

These leukocytes express a limited repertoire of germline-encoded receptors called pattern recognition receptors (PRR) recognizing conserved molecular motifs on the pathogens called pathogen associated molecular patterns (PAMPs)^29,30,34,35^. Moreover, the PRR can also sense the damage-associated molecular pattern (DAMPs) released by host cells experiencing trauma related or not to infection^30,35^. Altogether, these first defense lines constitute the innate immune system which is by nature fast acting but not specific^29,30,35^. The detection of PAMPs activates tissue-resident macrophages leading to the production and secretion of the pro-inflammatory cytokine interleukin 1 (IL1) through the formation and activation of the inflammasome, a large protein complex, at the interface of the mitochondria and the ER. This indicates that MERCs play a role in the development of the innate immune response^36–40^. Together, this leads to a state of inflammation in order to alert and to combat the ongoing infection.

Importantly, the innate immune system delivers the antigenic information to activate the adaptive immune system synergizing with the innate response. This adaptive response relies on the T lymphocytes (effectors of the cellular adaptive response) and the B lymphocytes (the antibody producing cells) harboring receptors encoded by genes requiring somatic rearrangements to be expressed^31,41,42^. Consequently, the adaptive response takes time to build-up and comes chronologically after the innate response^29,31^. Interestingly, the lymphocytes activation initiates a phosphorylation cascade resulting, among other things, in the mobilization of the intracellular Ca^2+^ pool essential for gene expression^43–47^. The ignition and development of both an innate and an adaptive immune response require immune cell activation, phagocytosis and processing of pathogens, migration, proliferation, release of soluble factors, and finally, the destruction of the infected cells. Some of these functions are strongly dependent on lipid transfer, autophagosome formation, mitochondrial fission and Ca^2+^ flux indicating that MERCs could regulate immunity.

Recent excellent reviews have discussed in great detail the MERCs molecular players and functional implications for Ca^2+^ and lipid transfer, as well as the mitochondrial metabolism; therefore, this subject will not be addressed here^4,6,15,27^. Instead, in this review, it will be put into perspective how the different function of the MERCs could impact on critical steps of both innate and adaptive immunity and see whether future work could also focus on the MERCs to regulate the host immune system.

## MERCs in innate immunity

The immune system is essential for human survival as in its absence even a minor infection can be lethal^48–50^. Once the physical barrier is breached, microbes access the host organism initiating the activation of innate immunity. Thanks to their toll-like receptors (TLR), a subfamily of PRR, patrolling macrophages are quickly alerted to the invasion^32,51–54^, which initiates the innate immune response and brings a state of inflammation^55^.

**Fig. 1:**
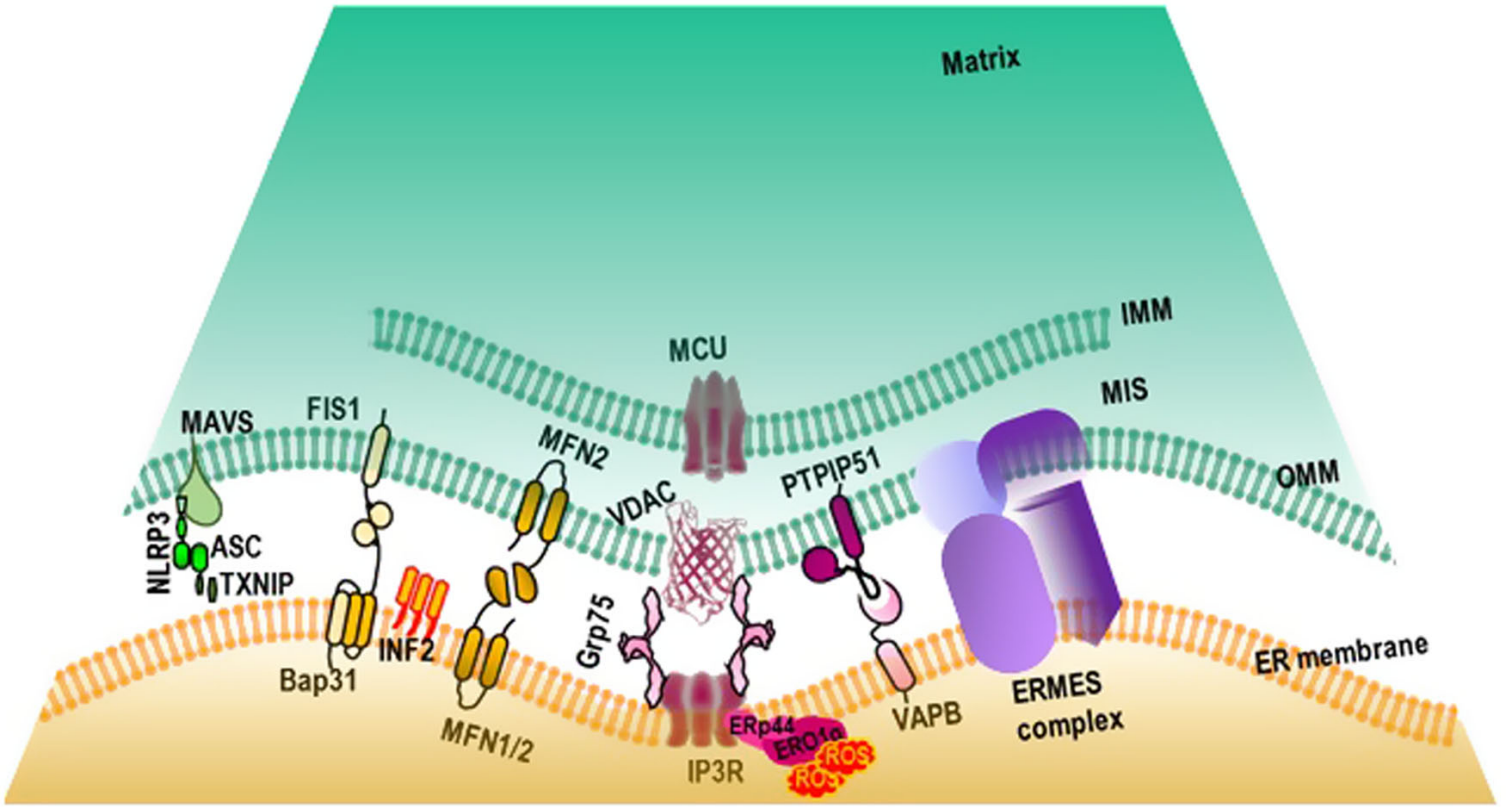
Graphic representation of the mitochondria-ER contact sites (MERCs) highlighting the principal molecular tethers. Mitochondrion (green) is juxtaposed with the endoplasmic reticulum (ER) (orange) forming an area of contact stabilized by molecular tethers, the nature of which determine the MERCs function. Matrix, mitochondrial matrix; IMM, inner mitochondrial membrane; MIS, mitochondrial intermembrane space; OMM, outer mitochondrial membrane; MCU, mitochondrial calcium uniporter; VDAC, voltage-dependent anion channel; Grp75, 75 kDa glucose-regulated protein; IP3R, inositol 1,4,5 three phosphate receptor; VAPB, vesicle-associated membrane protein-associated protein B; PTPIP51, protein tyrosine phosphatase-interacting protein 51; ERMES, ER-mitochondria encounter structure complex; MFN1 and 2, mitofusin 1 and 2; INF2, inverted formin 2; FIS1, Mitochondrial fission 1; Bap31, B-cell receptor-associated protein 31; MAVS, Mitochondrial antiviral-signaling protein; NLRP3, NOD-like receptors P3; TXNIP, thioredoxin-interacting protein; ASC, apoptosis-associated speck-like protein containing a CARD; ERp44, Endoplasmic reticulum resident protein and ERO1α, oxidoreductin 1 α.

This inflammation is triggered by the engagement and the activation of NLRP3 which belongs to the nucleotide-binding oligomerization domain-like receptors (NLRs)^56^, a subfamily of cytosolic PRRs, particularly potent at inducing inflammation following a wide range of stimuli, such as ATP^57^, hyaluronan^58^, uric acid crystal and amyloid-β^59^. One common feature of these triggers is their ability to induce reactive oxygen species (ROS) production, suggesting that ROS are critical for NLRP3 activation^56,60,61^. The NLRP3 activation and oligomerization recruit the adaptor protein apoptosis-associated speck-like protein containing a CARD (ASC) and procaspase 1 to form the high-molecular weight inflammasome protein complex^56–61^. The inflammasome is in fact a platform for caspase 1 activation and caspase 1-mediated processing of pro-IL1β and IL18^56,57^. The implication of ROS in NLRP3 activation suggests the involvement of MERCs in inflammation^40^. In unstimulated cells, NLRP3 is associated with the ER, while upon activation, it redistributes to the perinuclear region at the contact site between mitochondria and the ER (Fig. 1)^40^. Moreover, oxidation of active site cysteine thiols of thioredoxin (Trx) leads to the dissociation of thioredoxin-interacting protein (TXNIP) from Trx^39^ which binds to NLRP3 and robustly activates the inflammasome^39^. Interestingly, both ASC and TXNIP accumulate at the MERCs upon ROS-dependent activation (Fig. 1)^36–38,40^. In fact, TXNIP expression is induced by ER stress through the protein kinase R (PKR)-like endoplasmic reticulum kinase (PERK) and inositol-requiring enzyme 1 (IRE1) pathways which enhance IL1β expression followed by its maturation through NLRP3 inflammation^36,62^. Since PERK is also a MERCs tether, whether its contribution resides in its tethering function or its ability to induce TXNIP expression needs clarification. Similarly, whether the modulation of the MERCs could hinder the activation of NLRP3 inflammasome is an interesting question that needs further consideration. In agreement with this possibility, silencing of the three human isoforms of the voltage-dependent anion channel (VDAC), another MERCs tether, severely reduced inflammasome activation and IL1β production^40^. This result could also be explained by the contribution of VDAC in mitochondrial Ca^2+^ overload and ROS production, the latter being an agonist of NLRP3-inflammasome. VDAC is also a docking factor for mitochondria-interacting proteins, such as the metabolic switch enzyme hexokinase and the anti-apoptotic protein Bcl-2 and Bcl-X_L_ providing the cells with both metabolic advantage and resistance to apoptosis. This resistance to cell death results from the obstruction of VDAC channel cutoff by Bcl-2 and Bcl-X_L_ decreasing mitochondrial Ca^2+^ entry and ROS production necessary for NLRP3 inflammasome activation and IL1β production^37,63,64^. However, VDAC also interacts with Grp75 and the inositol-1,4,5-triphosphate receptor (IP3R) to actually physically tether the mitochondria to the ER (Fig. 1). Therefore, it is possible that silencing of VDAC also physically alters the localization and activation of the NLRP3-inflammosome at this site^40^.

Moreover, the identification of mitochondria antiviral-signaling protein (MAVS) as the docking site of the NLRP3 inflammasome at the MAM further confirmed the importance of MERCs in inflammation (Fig. 1)^65,66^. Interestingly, MAVS belongs to the mitochondrial antiviral response machinery involved in the production of type I interferons (IFNs) and pro-inflammatory cytokines^67^. In fact, upon infection, viral uncapped 5′-triphosphate end RNA and long double-stranded RNA are detected respectively by retinoic acid-inducible protein I (RIG-I) and melanoma differentiation-associated gene-5 (MDA-5)^68–70^, two cytosolic PRR with helicases and ATPase activity which discriminate the viral RNA from the abundant host RNA in the cytoplasm^71,72^. The binding of viral RNA triggers a conformational change in RIG I and MDA-5 that allows their interaction with MAVS at the MAM^65^. Their activation and translocation to the MERCs initiate signaling pathways that lead to the synthesis of multiple cytokines which include type I interferon (IFN)^65,67,73,74^. Interestingly, it was also reported that MAVS constitutively interacts with mitofusin 2 (MFN2), another MERCs tether, leading to the inhibition of inflammatory cytokine production. This suggests that the relocation of this antiviral response platform at the MERCs plays a complex regulatory role^72,75^ and as a whole, provide direct evidence of the involvement of MERCs in the establishment of a physiological inflammatory reaction as part of the innate immune response (Fig. 1 and Table 1). Further understanding of the spatiotemporal coordination of this inflammatory protein network and its regulation by MERCs is critical to therapeutically tailor inflammation as required.

**Table 1:** Immune involvement of the MERCs cell biology functions

		Innate immunity	Adaptive immunity
MERC functions	Ca^2+^ signaling	Leukocyte migration	Leukocyte migration
			Lymphocyte activation
			Sensitization to cell death, B and T cell homeostasis
	Cell death		B- and T-cell homeostasis
	Mitochondrial fission	Leukocyte migration	Leukocyte migration
	Inflammation/antiviral response	Initiation of the innate response	
	Autophagy		Antigen presentation, activation of T lymphocytes
	Lipid transfer		Modulation of the cytotoxic anticancer response

MERCs play many essential cell biology functions, e.g., calcium signaling, cell death, mitochondrial fission, inflammation, and antiviral response, lipid transfer and autophagy that are connected to immunological processes they are likely to regulate.

## MERCs and leukocyte migration

One key features of leukocytes is their ability to migrate throughout the organism. To illustrate this point, inflammation recruits hordes of neutrophils which massively infiltrate the site of infection to kill the invading bacteria^76,77^. Moreover, the activation of resident macrophages and dendritic cells by the PAMP and DAMP increases their ability to migrate to the closest draining lymph node where they will present the antigenic material to the naive B and T lymphocytes^78–85^. Then, these activated lymphocytes migrate to the site of infection to neutralize the infected cells; therefore, migration is a necessity for the proper function of both innate and adaptive immune cells. Interestingly, leukocyte migration requires drastic reorganization of their cytoskeleton and mitochondrial network^86,87^.

Mitochondria are versatile organelles with a well-established role in cellular energy production and metabolism, Ca^2+^ homeostasis, cell cycle regulation, differentiation, cell death and aging^88–93^. Mitochondria are constantly remodeled by fusion and fission events which are regulated by a family of dynamin-related GTPases and their adaptor proteins. Mitofusin (MFN) 1 and 2 and optic atrophy 1 (OPA1) regulate outer and inner mitochondrial membrane fusion, respectively^94–100^. Mitochondrial fission is mediated by cytosolic dynamin-related protein 1 (DRP1) docking on its adaptor proteins Fis1, mitochondrial fission factor (MFF) and mitochondrial dynamics 51 and 49 kDa proteins (MiD51 and MiD49) on the outer mitochondrial membrane^101–107^. Mitochondria can respond to many cellular cues such as starvation, stress-induced depolarization and cell death^94,95,98,99,106,108–116^.

**Fig. 2:**
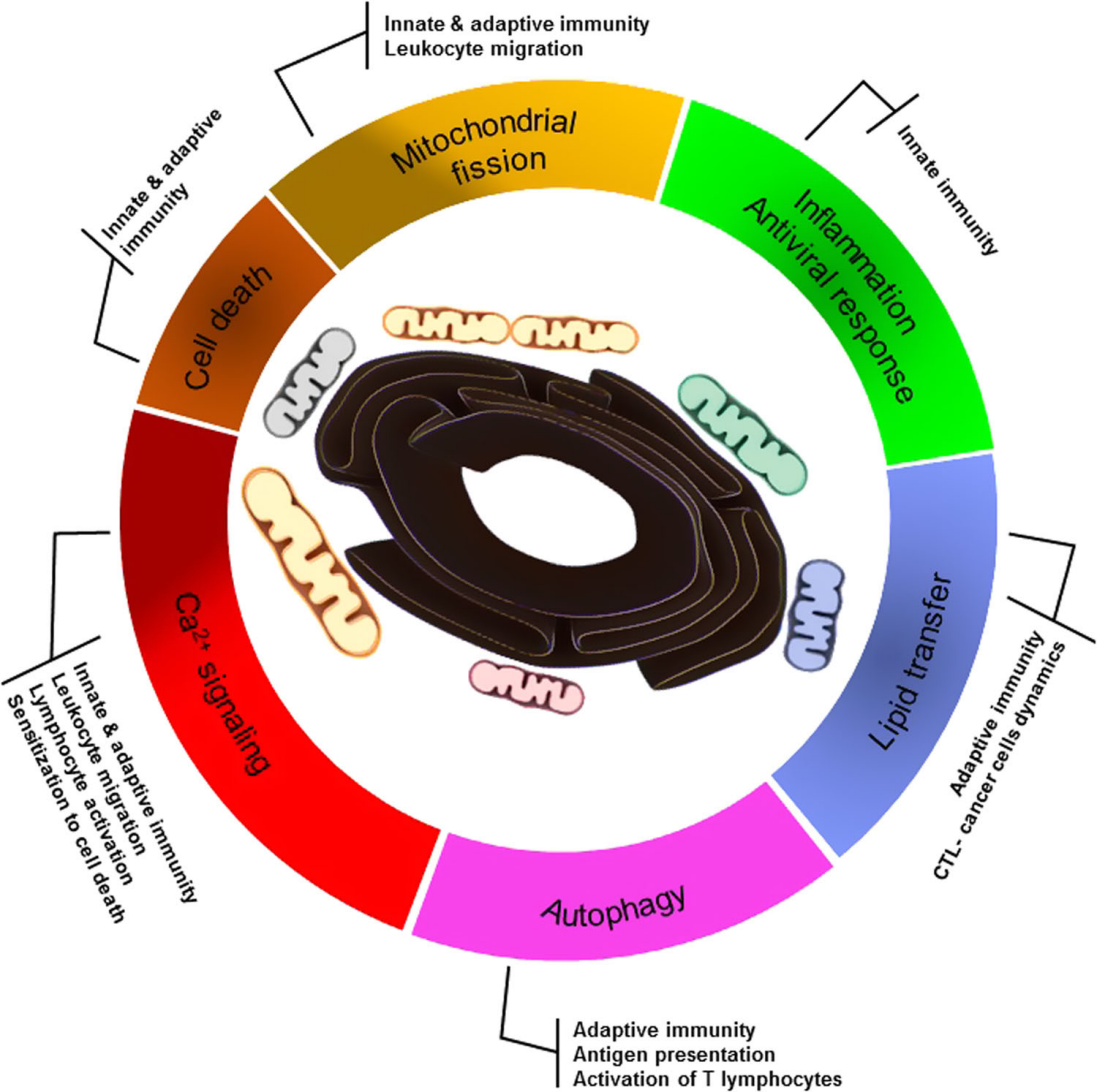
Wheel shaped representation of MERCs functions and their immunological involvement. In the center, ER (black) interacts with different shaped mitochondria to form MERCs. Wheel display of the corresponding cell biology functions involving the MERCs, e.g., calcium signaling (red), cell death (dark orange), mitochondrial fission (yellow), inflammation and antiviral response (green), lipid transfer (purple) and autophagy (pink). Note in the vicinity of the cell death segment, the segment representing calcium signaling and mitochondrial fission are darkened to indicate the gray area where calcium signaling and mitochondrial fission crosstalk with cell death. These MERCs cell biology functions are connected to the physiological immunological processes they are susceptible to regulate.

Interestingly, it was shown that during leukocyte migration there is a redistribution of the mitochondria at the cell uropods in a mitochondrial fission- and calcium-dependent manner^86,87,117,118^. Mitochondrial fission facilitates their relocation and promotes lymphocyte chemotaxis, whereas mitochondrial fusion inhibits both processes probably due to the inability to transport too large organelles along the cytoskeleton^86,87,117–119^. Interestingly, both mitochondrial fission and cellular Ca^2+^ homeostasis are regulated by MERCs (Figs. 1, 2 and Table 1)^11,14,18,20,22,27^. At the MERCs defined by the ER tubules wrapping the mitochondria, the mitochondria are constricted^3,11^. It was also proposed that these MERCs provide a platform for the recruitment of motor generating force cytoskeletal proteins^3^. In fact, ER-bound inverted formin 2 (INF2) concentrates between the two organelles where ER wraps the mitochondria (Fig. 1)^3,20,120^. The INF2 triggers the assembly of the actomyosin motor providing the force for the initial constriction of the mitochondria^3,11,20,120^. Once assembled, the ER-associated constricted mitochondria enable polymerized DRP1 to spiral around the mitochondria to mediate their fission^3,11,20,101,102,106,120^. Moreover, mitochondrial movement along microtubules is regulated by calcium oscillation-dependent Miro-Milton complex interaction with kinesin motor^117,121–124^. Interestingly, Miro is an outer mitochondrial membrane protein enriched at the MERC^123,125^. It interacts with dynein through the cytosolic factor Milton giving molecular insight into how calcium regulates the mitochondrial redistribution that also occurs during cell migration^122,126,127^.

Similarly, MERCs also play an important role in intracellular calcium homeostasis. Actually, the regulation of mitochondrial calcium uptake is the best described MERCs function so far. In resting condition, Ca^2+^ level ranges from nanomolar to micromolar concentration in the cytosol and the mitochondria, respectively, while it is in the half millimolar range in the ER^93^. This asymmetric calcium distribution is tightly regulated by a variety of calcium channels, pumps and exchangers expressed at the plasma membrane, the ER, and the mitochondria^14,22,43,46,47,128–134^. The coordination of the different calcium pools relies on membrane contact sites between the ER and the plasma membrane, and between the ER and mitochondria, thus acting as signaling platforms to ensure synchronized activities of Ca^2+^ channels, pumps and exchangers^3,12,43,46,47,128,131–134^. Upon activation, phospholipase C (PLC) produces inositol-1,4,5-triphosphate (IP3) from plasma membrane phosphatidylinositol-4,5 diphosphate^135,136^. The IP3 triggers ER Ca^2+^ release through a channel formed at the ER membrane by the IP3 receptor (IP3R)^135,136^. This IP3R-mediated ER calcium release is buffered by mitochondrial Ca^2+^ uptake through VDAC and the mitochondrial Ca^2+^ uniporter (MCU), located in the outer and inner mitochondrial membrane, respectively (Fig. 1)^131,132^. Both IP3R and VDAC are concentrated at ER-mitochondria contact sites where they also contribute to tether these two organelles together (Fig 1)^3,12^. The 75 kDa glucose-regulated protein (GRP75) interacts with both IP3R and VDAC to reinforce and increase the coupling of these two ion channels (Fig 1)^3,12,137^. This ER-mitochondria Ca^2+^ shuttling is regulated by a supramolecular weight protein complex including AKT kinase, promyelocytic leukemia (PML) and the serine threonine phosphatase PP2A enriched in the MAM^138–140^. Moreover, the vesicle-associated membrane protein-associated protein B (VAPB), an integral ER protein whose amino-terminus projects into the cytosol, interacts with the outer mitochondrial membrane protein tyrosine phosphatase-interacting protein 51 (PTPIP51) to favor the ER-mitochondria Ca^2+^ exchange (Fig. 1)^141^. The apposition of the ER with the mitochondria at these contact sites forms a confined space enabling to build-up local Ca^2+^ microdomains. These Ca^2+^ microdomains reach concentration compatible with MCU’ low affinity in order to ensure mitochondrial calcium uptake. This mitochondrial calcium uptake is necessary for activation of TCA dehydrogenase involved in ATP production and in the regulation of cell death^28,142–146^. Together, this indicates that MERCs, by their ability to regulate mitochondrial fission and calcium homeostasis, can modulate leukocyte migration and function (Table 1 and Fig. 2).

## MERCs and antigen presentation

Dendritic cells (DCs), macrophages and B cells are professional antigen presenting cells (APC) as they excel in the ability to ingest and process antigenic material to present in the context of their major histocompatibility complex class I (MHC-I) or class II (MHC-II) molecules in order to activate cytotoxic CD8^+^ T cells or CD4^+^ helper T cells, respectively^78,79,147–149^. The mechanism of antigen presentation has been reviewed in detail elsewhere^148,149^. Conventionally, MHC-I molecules are normally loaded with peptides derived from cytosolic proteolysis, while MHC-II molecules are loaded with peptides from extracellular pathogens that have been phagocytosed^148,149^. When DCs are directly infected with viruses, they generate MHC-I antigenic peptides by the classical pathway. However, in situations where the DCs are not directly infected or in case of tumors and allogeneic transplants, the antigens are internalized by phagocytosis of microbes, infected, allogeneic or transformed dying cells and cross-presented on the MHC-I of the DCs^148–151^. Interestingly, autophagy potentiates both MHC-I, MHC-II antigen presentation and MHC-I cross-presentation, the latter being seen when the autophagic process is triggered in donor cells^152–160^. Autophagy is also critical for the survival, differentiation and function of T lymphocytes and therefore plays an important role in the immune response^161,162^. There are at least three different types of autophagy, including macroautophagy (usually simply referred as autophagy), chaperone-mediated autophagy and micro-autophagy^163^. Autophagy is the process by which cytosolic components and organelles are segregated in a double membrane neocompartment, the autophagosome, for degradation and recycling following autophagosome fusion with lysosomes^157,164^. Nutrient deprivation is a potent inducer of autophagy through the inhibition of the mammalian target of rapamycin (mTOR)^165,166^. This leads to the activation and relocation of mTOR substrates ULK1/2, ATG13 and FIP200 from the cytosol to certain domains of the ER and the subsequent recruitment of the class III phosphatidylinositol kinase (class III-PI3K) complex VPS34/VPS15/beclin 1 and ATG14 to the ER^163,167–171^. The autophagosome formation also requires ATG12-ATG5 and the phosphatidylethanolamine (PE)-conjugated ATG8/LC3, GATE16, and GABARAP two ubiquitin-like conjugating systems^163,172–176^. It is suggested that the ER is crucial for the initiation of the autophagosome formation which takes place at the contact site between the ER and the mitochondria^19,177^. Moreover, upon starvation, the pre-autophagosome marker ATG14 is redistributed at MERCs while ATG5 also localizes at this site until autophagosome formation is completed^19^. Strikingly, starvation also triggers an increase in MERCs length^17^. Whether this increase in MERCs length could be a mechanism to modulate autophagy and the dependent antigen presentation needs to be addressed. Taken together, the critical importance of autophagy during antigen presentation could suggest that MERCs are likely critical regulators of lymphocyte activation as they contribute to autophagosome biogenesis (Table 1 and Fig. 2).

## MERCs and lymphocyte activation

**Fig. 3:**
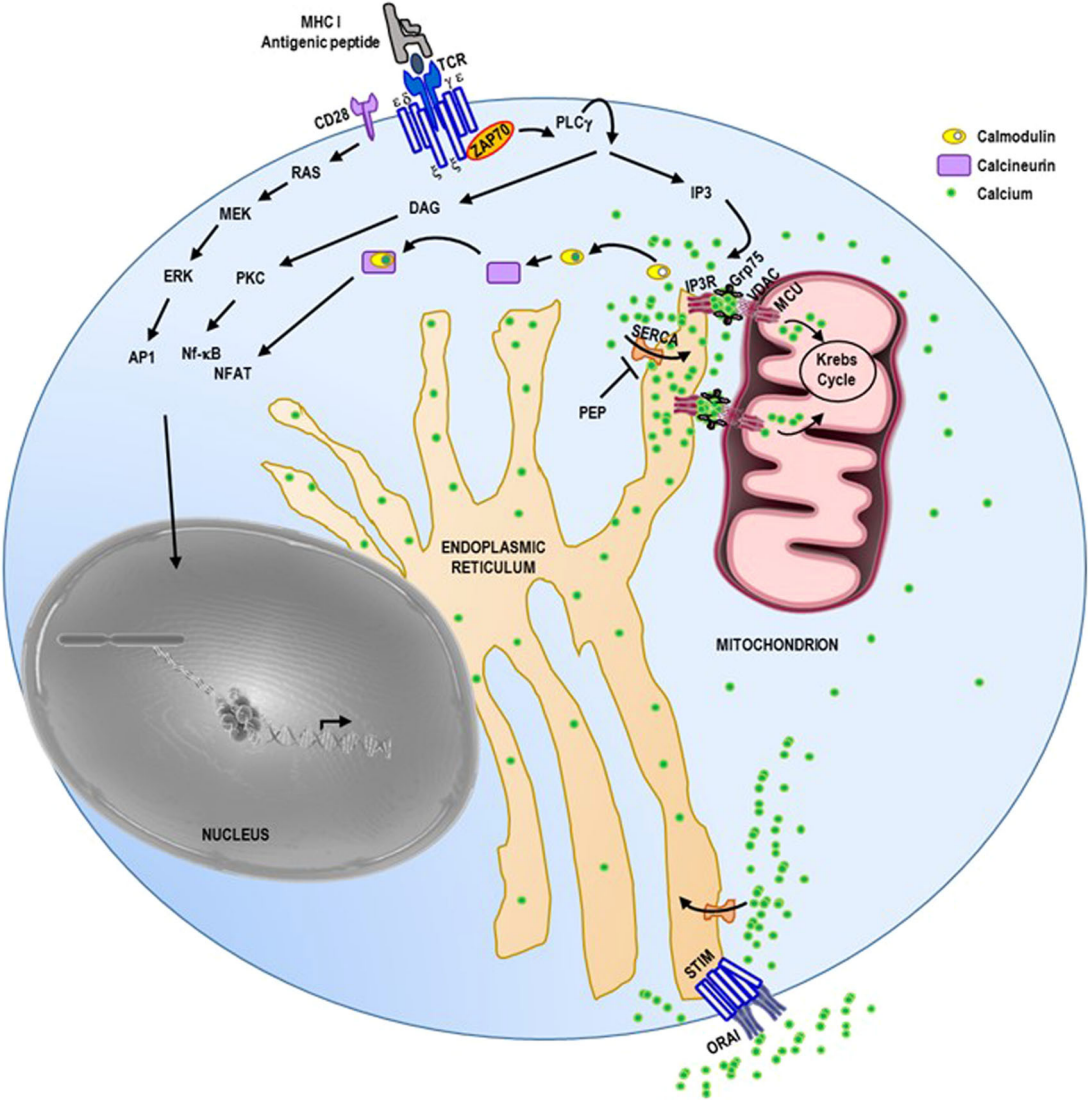
MERCs and calcium signaling during T cell activation. Cytotoxic T cells receive antigenic stimulation from MHC I antigenic peptide complexes at the surface of target cells (not represented) through its TCR CD3 complex. This triggers a signaling cascade inducing the activation of the kinase ZAP70 which activates phospholipase PLCγ. Active PLCγ hydrolyzes membrane phosphatidylinositol 4,5-bisphosphate into IP3 and diacylglycerol (DAG). DAG activates protein kinase C (PKC) which then activates the transcription factor NF-κB. IP3 binds to the IP3R on the endoplasmic reticulum (ER) to trigger calcium (Ca^2+^) release. In the cytosol, calcium binds to calmodulin and the complex Ca^2+^/calmodulin activates the phosphatase calcineurin leading to the activation of the transcription factor NFAT. The engagement of CD28 co-receptor at the surface of the T lymphocyte will trigger RAS, MEK, and ERK kinase cascade ultimately leading to AP1 transcription factor activation. The NFAT, NF-κB and AP1 enter the nucleus where they activate the expression of a subset of genes encoding for proteins involved in T lymphocyte proliferation, differentiation and effector function. The ER calcium depletion is sensed by STIM that in turn activates the plasma membrane calcium channel Orai which allows extracellular calcium to enter the cytosol. The ER calcium pump SERCA will pump calcium from the cytosol back into the ER. The MERC tether IP3R/VDAC/Grp75 allows the formation of microdomains of high-Ca^2+^ concentration for mitochondrial uptake through the mitochondrial calcium uniporter (MCU). In this case, this mitochondrial calcium uptake, by buffering the cytosolic calcium, may modulate the activation threshold of the T lymphocyte. Likewise, by inhibiting SERCA, phosphoenol pyruvate (PEP) reduces T lymphocyte activation threshold. ε, δ, γ, ξ are the subunits of the CD3 complex providing signaling module to the T-cell receptor.

Engagement of the B-cell receptor (BCR) or the T-cell receptor (TCR) and their respective co-receptors triggers an intracellular phosphorylation cascade culminating in the activation of the transcription factors AP1, NF-κB, NFAT, OCA-B/OBF-1 and Pip/IRF-4 that are critical for B and T lymphocyte activation, respectively^31,178–182^. One common feature of lymphocyte activation is the recruitment and activation of phospholipase Cγ (PLCγ) (Fig. 3)^31,179,180^ which mediates the production of IP3 and diacylglycerol (DAG). The DAG activates protein kinase C (PKC) initiating a phosphorylation cascade ending with the activation of the transcription factor NF-κB (Fig. 3)^183–186^. The IP3 induces a rapid increase in intracellular Ca^2+^ levels by means of activation of stores operating calcium (SOC) influx following IP3R-mediated ER calcium store release and activation of calcium-release activated calcium (CRAC) channel^43,46,47,133–136^. Cytosolic Ca^2+^ binds to calmodulin to activate the phosphatase calcineurin. Active calcineurin/calcium/calmodulin complex dephosphorylates and activates the transcription factor nuclear factor of activated T cells (NFAT)^31,44^. Since intracellular Ca^2+^ flux is essential for lymphocyte activation, any biological processes impeding on this Ca^2+^ homeostasis can impact lymphocyte activation. For example, phosphoenol pyruvate (PEP), by repressing the activity of sarco/ER Ca^2+^-ATPase (SERCA) whose function is to pump Ca^2+^ back into the ER, sustains TCR-mediated Ca^2+^-NFAT signaling and potentiates T cell activation^187,188^. Interestingly, mitochondria buffer the inflowing Ca^2+^ and prevent the blunting of the CRAC Ca^2+^ allowing full activation of T cells^87,189^. As discussed earlier, MERCs are particularly involved in the modulation of the cellular Ca^2+^ homeostasis; this suggests that MERCs most likely play a critical role in modulating lymphocytes activation (Table 1 and Figs. 2, 3). As already stated, mitochondrial Ca^2+^ overload sensitizes cells to death (Fig. 2 darkened zone of the calcium signaling segment)^22,146,189–193^. Furthermore, the interaction of the outer mitochondrial membrane protein Fis1 with the ER protein Bap31 constitutes an additional MERC tether, whose function is to provide a platform for apoptosis induction (Fig. 1)^28,194–196^. This suggests that MERCs could also act on lymphocyte homeostasis by regulating their development during negative selection and their elimination after antigen-dependent peripheral expansion in the process called activation induced cell death (AICD) (Table 1 and Fig. 2). Both processes involve massive lymphocyte death^42,197–199^.

## MERCs and the cytotoxic anticancer response

The role of the immune system against cancer was only demonstrated as primary and acquired immunodeficiency are associated with increased susceptibility to cancer^200–203^. Furthermore, the size of the immune infiltrate in primary tumor is a good prognosis for patient survival, explaining why blockage of immune checkpoint receptors CTLA4 and PD-1 is a very promising immunotherapy strategy^202,204–208^. Further supporting the importance of the anti-tumoral immunity, it was shown that among 810 mutant mouse lines screened to identify microenvironmental regulators of metastatic colonization, only 23 genes were important among which 19 have immune function^209^. Nevertheless, the occurrence of cancers is a direct demonstration that tumor cells are capable of evading the immune surveillance^202,210–213^. This ability to escape immune recognition and elimination is now a new hallmark of cancer^214^.

**Fig. 4:**
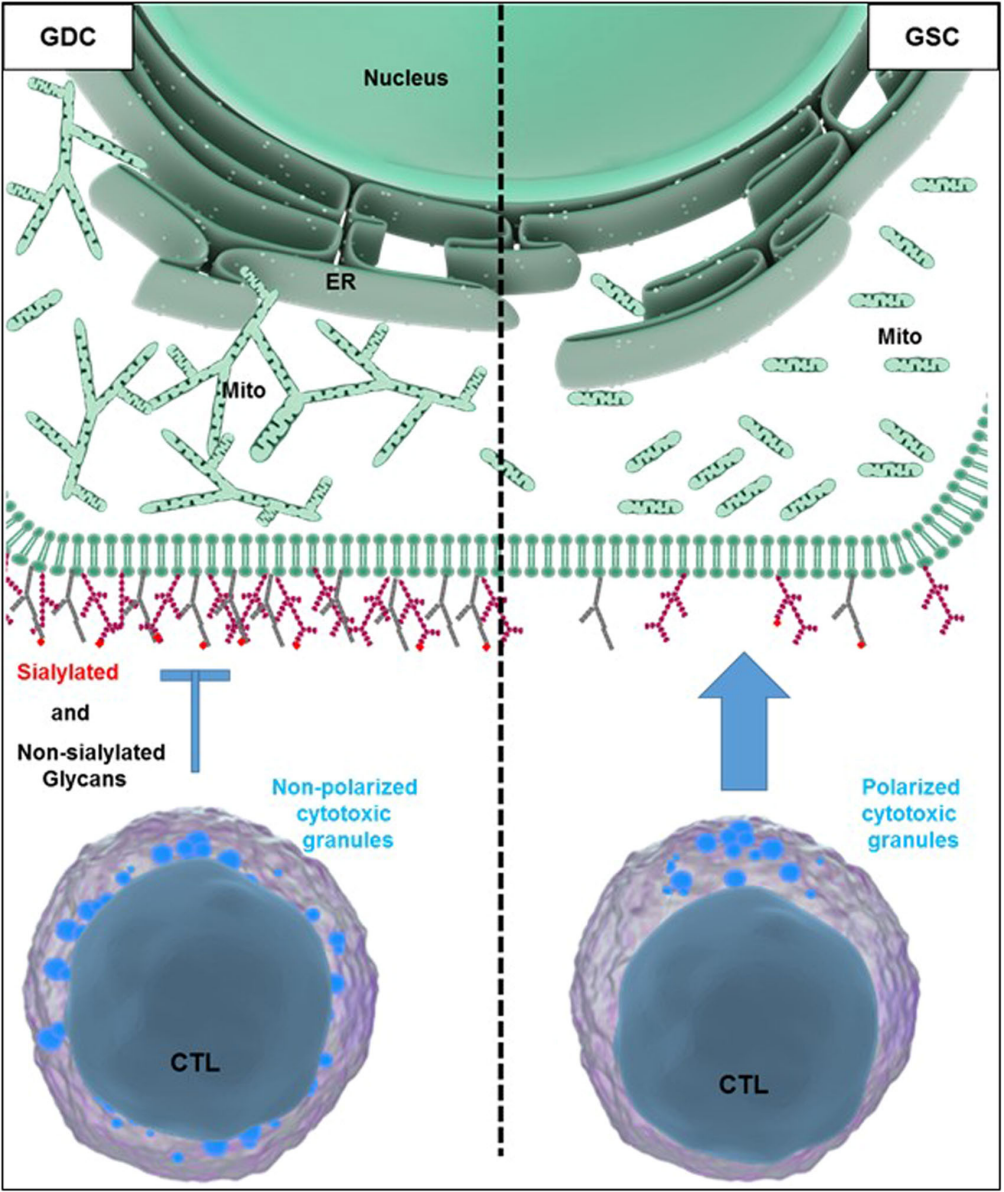
Mitochondria morphology and dynamism regulate glioma surface glycan expression and sensitivity to cytotoxic lymphocytes mediated killing. On the left, glioma differentiated cells (GDC) have reticulated long mitochondria that interact well with the ER to form MERCs, leading to a high-surface expression of some glycans, impeding engagement and killing by cytotoxic lymphocytes. On the right, glioma stemlike cells have short and highly dynamic mitochondria that make less MERCs, resulting in a lower surface expression of glycans, better engagement and killing by cytotoxic lymphocytes. ER, endoplasmic reticulum; Mito, mitochondria

Strikingly, cancer cell plasma membrane topography and glycocalyx regulate the ability of cytotoxic lymphocytes to contact them^215,216^. To trigger apoptosis, cytotoxic lymphocytes must form an immunological synapse with their cancer target cells into which they degranulate their cytotoxic granule content^45,179,217–223^. We found this pathway to also be dependent on target cell mitochondrial ROS production^219–222,224^. Using a glioblastoma multiform model, a very aggressive primary malignant brain tumor, we found that surface glycan expression regulates these cancer cells engagement by cytotoxic lymphocytes^225,226^. In fact, the glioma stemlike cells (GSC) which expressed lower surface sialylated glycans were more susceptible to cytotoxic lymphocytes as opposed to the glioma differentiated cells (GDC) (Fig. 4)^226–231^. Compared to GDC, DRP1 expression was higher in GSC, while MFN2 expression was reduced, explaining their fragmented mitochondrial phenotype^226,232^. MFN2 was the only MERCs tether whose expression was significantly reduced in GSC as opposed to GDC^28,137,141,233,234^, in agreement with GSC shorter mitochondria poorly interacting with the ER, than did the elongated mitochondria in GDC. Consequently, after stimulation, GSC experienced reduced mitochondrial Ca^2+^ uptake as compared to their GDC counterparts. Excitingly, restoring the ER-mitochondria contact in GSC with an artificial tether was sufficient to restore the surface expression of certain sialylated glycans and reduce their susceptibility to cytotoxic lymphocyte-mediated killing (Fig. 4)^226^.

Protein and lipid glycosylation are critical for cell physiology^235–237^. Changes in glycosylation and the expression level of surface sialic acid and sialyltransferase are directly correlated with tumor metastasis^238–240^. Interestingly, MAM is enriched in glycosyltransferases and ceramide synthase activities^241–243^. Moreover, in addition to the vesicular transport system, the exchanges and the biosynthesis of lipids also require MERCs^3,21,244,245^. In yeast, the ERMES complex at the MERCs is composed of Mmm1, Mdm10, Mdm12, and Mdm34 that are functionally connected to phospholipid biosynthesis^21,244,246^. During their biosynthesis, lipids commute back and forth between ER and mitochondria at membrane contact sites. In the ER, phosphatidic acid is converted to phosphatidylserine which is further decarboxylated in the mitochondria inner membrane to form phosphatidylethanolamine. Phosphatidylethanolamine shuttles back to the ER where it is transformed into phosphatidylcholine^3,12,23,247^. Therefore, it is likely that the MERCs defects observed in GSC could result in an altered biosynthesis or bioavailability of lipids, essential for the surface expression of some glycolipids. In future studies, it would be very important to test whether MERCs dysregulation is a novel feature of cancer stemlike cells regardless of their histological origin.

## MERCs and human diseases

MERCs may be a novel regulatory hub for cancer development through the recruitment of proto-oncogenes and tumor suppressor^15^. Indeed, extra nuclear accumulation of the tumor suppressor PML at the MERCs, where it forms a supramolecular weight complex with PP2A and AKT, provides a regulatory module for the ER-mitochondrial Ca^2+^ transfer apparatus^140,248^. This localization of a pool of PML is essential for the propagation of apoptotic stimuli following mitochondrial Ca^2+^ overload in conditions of cellular stress^140,248^. Similarly, PTEN, another tumor suppressor, is also enriched at MAM where it also regulates AKT-dependent phosphorylation of IP3R and ER-mitochondria Ca^2+^ shuttling. Interestingly, at the MERCs, mTORC2 interacts with the IP3R/Grp75/VDAC1 to regulate not only MAM’s integrity but also mitochondrial ATP production in a manner that relies on Akt-mediated phosphorylation of IP3R, Hexokinase 2, and phosphofurin acidic cluster sorting protein 2 (PACS2) equipping the MERCs with control over cellular growth and metabolism^15,249^. Interestingly, PACS2 which is also a MERCs tether, is mutated in 40% of sporadic colorectal cancer patient^250,251^.

Correct protein folding in the ER needs proper disulfide bond formation by oxidoreductin 1 (ERO1) α and protein-disulfide isomerase (PDI)^252^. Interestingly, ERO1α interacts with ERp44, a negative regulator of IP3Rs (Fig. 1) and ERO1α expression is independently regulated by both hypoxia and hypoglycemia, two known microenvironmental factors associated with cancer development^253,254^. In the same manner, it was also elegantly demonstrated in vivo using a postprandial model that inhibition of mTORC1signaling pathway leads to a doubling in MERCs length^17^; this indicates that nutrient abundance can regulate all the cellular functions where MERCs play a role. These results explain how cancer cells, by outcompeting tumor infiltrating T lymphocyte for glucose in the microenvironment can both inhibit the anti-cancer cytotoxic immune response while adapting to this microenvironment for cancer progression^17,187,188^. As a whole, these results explain how the tumor microenvironment could impede the structure and function of MERCs to promote cancer progression. Moreover, since MERCs regulate ER-mitochondria Ca^2+^ interplay and apoptosis, it is very likely that MERCs could also modulate cancer cells sensitivity to chemotherapy and the co-occurrence of the beneficial immunogenic cell death (ICD)^255^. The ICD also relies on the ability of dendritic cells to present antigen from the dying tumor cells to T-lymphocyte populations (see MERCs and antigen presentation). So, whether through the modulation of sensitivity to, type of cell death or through antigen presentation, MERCs are likely involved in the eradication of cancer cells.

MERCs regulate many important biological processes, the dysregulation of which can severely affect cell homeostasis and consequently human health. Inflammation is a good illustration of such a process through which MERCs alteration may contribute to other human diseases, such as neurodegeneration, diabetes, and cardiovascular diseases^4,13,15,256,257^. For more information I invite the readers to PMID:25557408; 24642268, and 24316057.

## Conclusion

The necessity to isolate biochemical reactions impose the cell compartmentalization with the consequence of restricting the mutualisation of essential constituents. To overcome this restriction, the different cell compartments communicate at a contact zone, defined as organelle membrane contact sites. The ER is the largest cell network that not surprisingly touches many other organelles, including the mitochondria. Mitochondria-ER contact sites (MERCs) play critical functions, such as lipid transfer, initiation of autophagosome formation, determination of the mitochondrial fission site, mitochondrial Ca^2+^ homeostasis, as well as apoptosis^11,14,18–28^. As we have seen, many of these functions are important for both innate and adaptive immunity. Actually, because of the different challenges immune cells must face to protect the organism from invading pathogens, they represent good models to further investigate the MERCs structurally, biochemically, and functionally. These investigations are expected to provide additional important insight into the role of these dynamic membrane interactions in the pathophysiology of the immune system.
